# ADVERSE CHILDHOOD EXPERIENCES AND ALCOHOL-IMPAIRED DRIVING IN LATER LIFE

**DOI:** 10.1093/geroni/igz038.2549

**Published:** 2019-11-08

**Authors:** Shalini Sahoo, Paul Sacco

**Affiliations:** University of Maryland-Baltimore, Baltimore, Maryland, United States

## Abstract

Adverse Childhood Experiences (ACEs) are associated with a range of negative behavioral health outcomes in adulthood. Those with ACEs may use alcohol as a component of long-term coping, increasing risk of alcohol-impaired driving. Associations between ACEs and alcohol-impaired driving are relatively understudied. Using the 2012/2013 data from the National Epidemiologic Survey on Alcohol and Related Conditions (NESARC), logistic regression models examined the relationship between five types of ACEs (e.g. child abuse and neglect) and lifetime alcohol-impaired driving among a representative sample of American adults aged between 18 to 90 years (N = 36,309). ACEs were positively associated with lifetime alcohol-impaired driving for adults under age 50 (witnessed intimate partner violence (OR = 1.624, p < 0.001), physical abuse (OR = 1.723, p < 0.001), sexual abuse (OR = 1.651, p < 0.001), physical neglect (OR = 1.571, p < 0.001), and emotional neglect, (P > 0.05). We found similar positive associations between ACEs and impaired driving among adults aged 50 and over (witnessed intimate partner violence (OR = 1.398, p < 0.05), physical abuse (OR = 1.751, p < 0.001), sexual abuse (OR = 1.690, p < 0.001), physical neglect (OR = 1.455, p < 0.001), and emotional neglect, p > 0.05). Among adults under age 50, ACEs were associated with past-year alcohol-impaired driving, but this relationship was not seen in adults aged 50 and over. Findings suggest that the effect of ACEs on alcohol-impaired driving is in younger adulthood when alcohol use and driving occur most.

